# A silent pandemic of violence against providers in obstetrics and gynecology: A mixed‐methods study based on a global survey

**DOI:** 10.1002/ijgo.15985

**Published:** 2024-11-02

**Authors:** Margit Endler, Atziri Ramirez‐Negrin, Rubina Sohail, the FIGO Committee on Women Facing Crises

**Affiliations:** ^1^ Department of Women's and Children's Health Karolinska Institutet Stockholm Sweden; ^2^ Department of Public Health University of Cape Town Cape Town South Africa; ^3^ FIGO Committee on Women Facing Crises London UK; ^4^ Department of Urogynecology Hospital Dr Manuel Gea Gonzalez Mexico City Mexico; ^5^ Hameed Latif Hospital affiliate of Rashid Latif Medical University Lahore Pakistan

**Keywords:** healthcare providers, obstetrics and gynecology, sexual and reproductive health and rights (SRHR), violence, workplace violence

## Abstract

**Objectives:**

To quantify and qualify the experience of workplace violence (WPV) in a global sample of providers in obstetrics and gynecology (OBGYN).

**Methods:**

We performed a mixed‐methods analysis on data from a global survey. Survey content was designed around categorical and open‐ended questions in relation to WPV; the occurrence and character, the physical and psychological consequences, training and support structures, and perceived triggers of the experience of WPV. Quantitative data were analyzed using descriptive statistics and text data using mixed deductive‐inductive content analysis. These data were integrated using convergent joint display.

**Results:**

Between October 2023 and January 2024, survey responses were collected from 77 individual countries. Among the final sample, 764/1016 (75.2%) had experienced WPV, 699/1016 (68.8%) verbal, and 123/1016 (12.1%) physical violence. The violence affected physical health, psychological health, or job satisfaction for 106/764 (13.9%), 36/7642 (47.4%), and 222/764 (29.1%) of individuals respectively; 216/764 (28.3%) received support. Main WPV triggers were staff shortages, lack of security personnel, and long waiting times, identified by 38.8%, 37.5%, and 37.3% of respondents respectively. Qualitative data indicated that violence caused severe and long‐lasting suffering. Catalysts for WPV were often reported as complex interplays between unmet or unrealistic expectations and insufficient resources. Lack of support for WPV was explained as violence being “part of the job” and a culture of assumed resilience among providers.

**Conclusion:**

WPV against OBGYN providers seems to be ubiquitous, arises from a complex interplay of factors, and causes significant injury while receiving insufficient mitigation and support.

## Synopsis

Global survey suggests that violence against OBGYN providers is pervasive, causes significant suffering, meets with insufficient support, and stems from a complex interplay of factors.

## 1 INTRODUCTION

Workplace violence (WPV) directed against healthcare providers is a major public health concern, of consequence not only to providers but to the healthcare system as a whole.^1^ According to WHO, WPV is an incident where staff are abused, threatened, or assaulted in circumstances related to their work, involving an explicit or implicit challenge to their safety, well‐being, or health.^2^

WPV against healthcare providers is common and seems to be increasing. A recent meta‐analysis of 253 studies including 331 544 participants found a mean 1‐year incidence of WPV of 61.8%.^3^ A 2018 survey conducted by the American College of Emergency Physicians demonstrated that out of more than 3500 emergency department (ED) doctors, 47% had been physically assaulted, in 97% of cases by a patient.^4^ In the USA, injuries caused by violent attacks against medical professionals grew by 67% between 2011 and 2018—with healthcare workers five times more likely to experience WPV than workers in all other industries.^5^ Data on violence against providers in the field of obstetrics and gynecology (OBGYN) is limited. A survey from 2018 among obstetricians and gynecologists in China found that two‐thirds had experienced violence while on duty in the previous year alone.^6^ Recent systematic reviews of the phenomenon of WPV against physicians indicate that its incidence varies between countries and contexts.^7^, ^8^ To our knowledge, no previous original study has had a global scope and collected qualitative data on healthcare providers' experiences of violence and the context within which it occurs.

The aim of the present study was to both quantify and qualify the life‐time experience of WPV in a global sample of providers in OBGYN.

## 2 MATERIALS AND METHODS

We performed a mixed‐methods study based on responses to an online survey sent out in October 2023. The survey content was developed through several iterations within the FIGO Committee on Women Facing Crises and directed primarily towards doctors in OBGYN.

The survey was translated into French, Spanish, and Arabic, and was available to attendants of the FIGO World Congress in Paris on October 9–11, 2023, through a QR code displayed at the welcome counter. The survey was subsequently emailed to all national societies of obstetrics and gynecology for dissemination.

The survey was constructed around four mixed research questions on the experience and perceptions of WPV:

1. What character of WPV do providers experience while on duty?
2. What are the physical and psychological consequences of experiencing WPV?
3. What contextual contributing factors for WPV do providers identify?
4. What support structures exist for providers that experience or are at risk of WPV and how are they perceived?

The survey contained 18 categorical questions on the type of violence experienced, impact on physical and psychological well‐being, impact on job satisfaction, situation triggers, and support and education structures within the healthcare system. These questions were paired with open‐ or close‐ended questions nuancing the categorical responses. For example, the question “Did the violence affect your emotional and psychological well‐being?” was followed by the question “How did the violence affect your emotional and psychological well‐being.” Here we used an integration strategy of matching to be able to integrate quantitative and qualitative findings.^9^, ^10^

Baseline characteristics were presented as absolute numbers and rates, and as relative rates according to whether the respondent had or had not experienced WPV. Associations between baseline characteristics age, gender, profession, length of professional experience, and region of residence were calculated using unconditional binomial logistic regression. Background variables with a significant association to having experienced violence were included in an adjusted model, unless they correlated too strongly with each other (*r* >0.6). Apart from region of work, potential confounders were suggested by previous studies.^3^

Text data were interpreted at the manifest level. The whole text was read through for familiarization. Within the limitations of using text data, we used a mix of deductive and inductive coding with survey questions/themes used as overarching codes and subthemes extracted within each theme. Three researchers (ME, RS, and AR) reviewed the text data.

We used mixed methodology to enhance the understanding of the experience of WPV. Quantitative and qualitative data were interpreted separately in parallel and then integrated using joint display in a convergent design where subthemes were assessed in relation to quantitative data for confirmation, complementarity, and contradiction.

Approval for the study was obtained from the Swedish Ethical Review Authority (reference no. 2020–04629).

## 3 RESULTS

**FIGURE 1:**
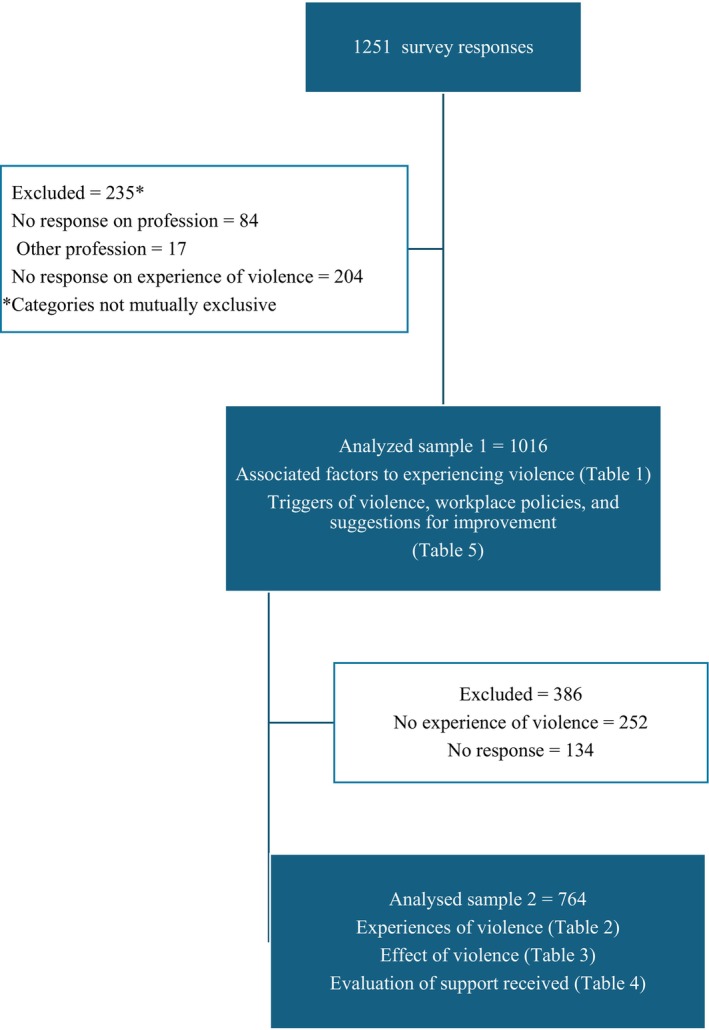
Inclusion into study of workplace violence against providers of obstetrics and gynecology.

Between October 9, 2023, and January 31, 2024, we collected 1251 responses from 77 individual countries. The total number invited to scan the QR code or who were sent the study through their national societies is not known, meaning that the response rate could not be calculated. We excluded respondents who reported a non‐medical profession (*n* = 17), who did not specify their profession (*n* = 84), or who did not respond to whether they had experienced violence (*n* = 204). Categories were not mutually exclusive and a total of 235 respondents were excluded. Our analyzed sample consisted of 1016 participants. We retained respondents who identified as “Other doctor,” as many were assumed to be under specialization in OBGYN or work closely aligned with OBGYN. Inclusion into the study is shown in Figure 1.

Among our respondents, 804 (75.1%) identified as female, 204 (20.1%) as male, and 1 (0.1%) as non‐binary. Specialists in OBGYN represented 781 (76.9%), while 235 (23.1%) were either nurse/midwives, doctors under specialization in OBGYN, or with an affiliated specialization, such as surgery or anesthesia. There was an even distribution among respondents in age and length of professional experience. Eight regions of the world were represented as follows: Africa (*n* = 149, 14.7%), Australasia (*n* = 5, 0.5%), Central Asia (*n* = 503, 49.5%), East Asia (*n* = 57, 5.6%), Europe (*n* = 88, 8.7%), the Middle East (*n* = 84, 8.3%), North America (*n* = 12, 1.2%), and South and Central America (*n* = 107, 10.5%).

**FIGURE 2:**
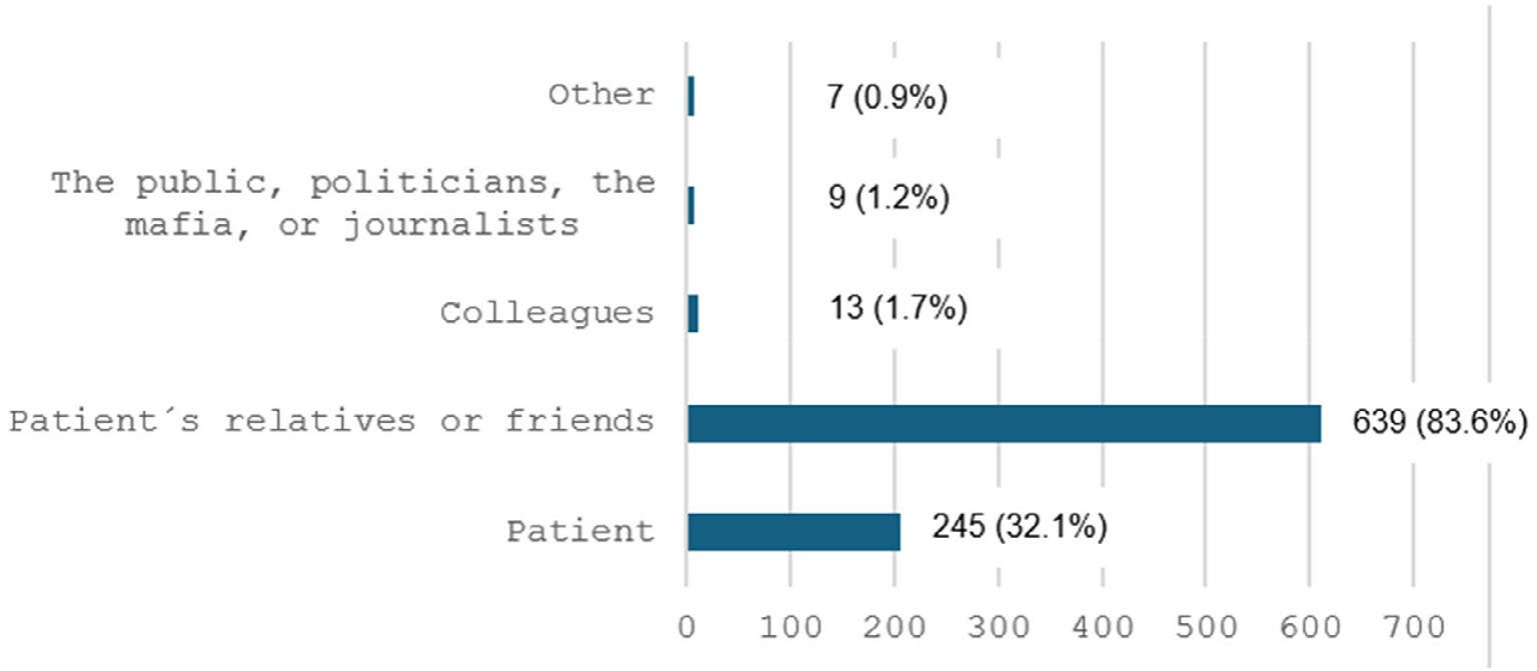
Reported perpetrator according to 764 doctors or nurses subjected to violence while on duty. Percentages exceed 100 since respondents may have experienced workplace violence from several different perpetrators.

Overall, out of 1016 respondents, 764 (75.2%) reported having experienced either verbal, physical, or another type of WPV. Gender and profession were not associated with an increased risk of violence, although men were more likely to have experienced physical violence (Tables 1 and 2). There was a linear increase in adjusted risk of having experienced violence with decreasing age and decreasing length of professional experience. Working in Central Asia, South or East Asia, Europe, or the Middle East was associated with a lower adjusted risk of having experienced violence. Baseline characteristics as well as associated risk of experiencing violence are presented in Table 2. The most common perpetrator according to our respondents was a relative of a patient (Figure 2).

**TABLE 1:** Background characteristics, rate, and risk of experiencing violence among 1016 providers in obstetrics and gynecology.^a^

	Experienced violence while on duty^b^			
Group characteristic	No	Yes	OR (95% CI)	Adjusted OR (95% CI)^c^
All participants	252 (24.8)	764 (75.2)		
Sex				
Female	199 (24.8)	605 (75.2)	ref	
Male	51 (25)	153 (75)	0.99 (0.69–1.41)	
Non‐binary	1 (100)	0 (0)	nc	
Prefer not to say	0 (0)	1 (100)	nc	
No answer	1 (16.7)	5 (83.3)	nc	
Profession				
OB/GYN	184 (23.6)	597 (76.4)	ref	
Nurse/midwife	13 (29.5)	31 (70.5)	0.96 (0.47–1.98)	
Doctor, other^d^	55 (28.8)	136 (71.1)	1.31 (0.92–1.87)	
Professional experience (years)				
0–10	61 (18.5)	269 (81.5)	**2.16 (1.51–3.08)**	**2.43 (1.66–3.54)**
11–20	73 (22.6)	250 (77.4)	**1.68 (1.19–2.36)**	**1.71 (1.19–2.44)**
>20	117 (32.9)	239 (67.1)	ref	ref
No answer			nc	nc
Region of work^e^				
Africa	29 (19.5)	120 (80.5)	0.59 (0.29–1.20)	0.47 (0.23–0.98)
Australasia	2 (40)	3 (60)	0.21 (0.33–1.41)	0.15 (0.02–1.00)
Central Asia	132 (26.2)	371 (73.8)	**0.40 (0.21–0.73)**	**0.30 (0.16–0.57)**
South/East Asia	23 (40.4)	34 (59.6)	**0.21 (0.10–0.46)**	**0.22 (0.10–0.50)**
Europe/Russia	25 (28.4)	63 (71.6)	**0.36 (0.17–0.76)**	**0.34 (0.16–0.72)**
Middle East	20 (23.8)	64 (76.2)	**0.46 (0.21–0.99)**	**0.42 (0.19–0.93)**
North America	2 (16.7)	10 (83.3)	0.71 (0.14–3.63)	0.73 (0.14–3.89)
South America	15 (14)	92 (86)	ref	ref
No answer	4 (36.4)	7 (63.6)	nc	nc
Age (years)				
≤30	22 (16.4)	112 (83.6)	**2.75 (1.51–5.00)**	**3.09 (1.67–5.72)**
31–40	63 (19.6)	258 (80.4)	**2.21 (1.38–3.55)**	**2.43 (1.49–3.98)**
41–50	60 (23.2)	197 (76.7)	**1.78 (1.10–2.87)**	**1.81 (1.11–2.98)**
51–60	62 (35.8)	111 (64.2)	0.97 (0.59–1.59)	1.00 (0.60–1.66)
>60	40 (35.1)	74 (64.9)	ref	ref

Abbreviations: CI, confidence interval; nc, not computed/not included in model; OB/GYN, obstetrics and gynecology; OR, odds ratio; ref, reference. Statistically significant effect measures are indicated in bold.

^a^ Values are given as number (percentage) unless otherwise indicated.

^b^ Incidence of physical violence was 18.1% (*n* = 37/204) for men and 10.6% (*n* = 85/804) for women (*P* = 0.003). For verbal violence, it was 65.2% (*n* = 133/204) for men and 70% (*n* = 563/804) for women (*P* = 0.183).

^c^ Adjusted for region and professional experience or age. Age and length of professional experience correlated strongly (*r* = 0.8) so are not included in the same model.

^d^ Includes doctors under OB/GYN specialization or specialist in related field, for example, surgery or anesthesia.

^e^ Respondents from Russia = 1. The South America category includes Central America (see Data S1).

**TABLE 2:** Rate and qualitative description of experience of violence among 1016 providers of obstetrics and gynecology while on duty.

Categorical type of violence and response options^a^		*n* (%)	Qualitative description of violence: samples
Verbal violence	Yes	699 (68.8)	*“Being yelled at, verbally abused, sworn at, because patients' unrealistic expectations with regards to appointments and availability were not met.”*
	No	252 (24.8)	*“If she dies, it's your fault!! You don't want to take care of me! I'm going to kill you and your entire family!!”*
	No answer	65 (6.4)	*“Racist rant and wanted a white doctor.”*
			*“Several situations but typically accused that we do not know how to do our job, that our care is not correct, that we are not attentive to our patients, that we are incapable.”*
Physical violence	Yes	123 (12.1)	*“Threatened with a gun while on night duty. Being abused while attending a patient.”*
	No	252 (24.8)	*“Punched by a trauma patient. Attacked by family and friends in theater 6 months ago after doing damage control surgery and opting to refer the patient out because their needs were beyond what we could offer.”*
	No answer	641 (63.1)	*“I have experience being slapped, hair pulled, scratched, kicked, punched, grabbed in a sexual manner, spit at, human waste thrown at me and have been verbally assaulted and threatened.”* *“I was beat up while working. It cost me everything and more.”*
Sexual or other violence	Yes	11 (1.1)	*“A patient was seriously ill and his relatives kept shouting at me, even threatened to take my video and make it viral.”*
	No	230 (22.6)	*“I was raped several times by a patient […] and I tried to ‘save myself’ and reject him while trying to not agitate him.”*
	No answer	775 (76.3)	*“Threatened by a patient's father to have me arrested by the police and locked up because she had a complication and adverse outcome*.*”*

^a^ The incidence of physical violence was 18.1% (*n* = 37/204) for men and 10.6% (*n* = 85/804) for women (age‐adjusted odds ratio [OR] 2.0, 95% confidence interval [CI] 1.29–3.12). For verbal violence, it was 65.2% (*n* = 133/204) for men and 70% (*n* = 563/804) for women (age‐adjusted OR 0.97, 95% CI 0.69–1.37). There were two reports of sexual violence, both from women.

Among all 1016 respondents, 669 (68.8%) had experienced verbal violence. Characterizations of verbal violence included shouting, insults, threats of physical violence including homicide, racism, and forceful accusations of incompetence, negligence, and responsibility for adverse outcomes, perceived to be outside the control of the provider. Many respondents reported frequent, sometimes daily, verbal abuse throughout their careers and abuse by large groups of people at a time. Frequently, respondents described disregard and lack of understanding for the context within which doctors had to operate:

*“If she dies, it's your fault!! You don't want to take care of me! I'm going to kill you and your entire family!!”* [OBGYN, Venezuela, translated from Spanish].

A smaller group (*n* = 123/1016, 12.1%) reported that they had experienced physical violence. Many respondents described being spit at, scratched, or slapped; however several described more severe assaults, such as being punched, held by the neck, kicks to the body or face, fractures, and threats at gunpoint.

*“I have experience being slapped, hair pulled, scratched, kicked, punched, grabbed in a sexual manner, spit at, human waste thrown at me, and have been verbally assaulted and threatened.”* (Midwife, USA).

A minority (*n* = 11/1016, 1.1%) reported having experienced what we characterized as another type of violence. Two respondents reported sexual violence, including rape. For most of these respondents, however, the violence consisted of threats of actions involving parties outside the patient–doctor relationship, such as the legal system, the media, or the police. The threat of social media was especially prominent.

*“A patient was seriously ill and his relatives kept shouting at me even threat(e)ned to take my video and make it viral.”* (Doctor, Pakistan).

A joint display of quantitative and qualitative data for the occurrence and characterization of violence is shown in Table 2 

Among the 764 providers who had experienced violence, 106 (13.9%) described a transient or long‐lasting physical impact. In the text data, some respondents described wounds or fractures needing to heal; however, more often, the violence was associated with chronic pain, migraine or tension headache, or the advent of chronic disorders, such as high blood pressure or autoimmune disease.

*“Chronic pain is exhausting, I never hurt like I do now 3 years later.”* (OBGYN, Colombia).

More respondents (*n* = 362, 47.4%) reported the violence impacting their psychological health and well‐being. Characterizations of the psychological impact of violence in the text data confirmed and complemented these findings with many respondents describing anxiety, depression, and insomnia. Many respondents further described fear of facing similar clinical scenarios, permanently losing empathy for patients, and becoming despondent and withdrawn from friends and family. Some respondents described severe psychiatric illness: deep depression requiring hospitalization, medication or therapy; acute stress disorders; symptoms suggesting post‐traumatic stress disorder; suicidal ideation or attempt; and self‐harm.

*“I lost sympathy with patients and (their) relatives. Lost interest in dealing patients. Felt disgusting. Cannot focus on professional work. Depression for the whole family.”* (Doctor, Pakistan).

Close to one‐third of the 764 respondents (*n* = 222, 29.1%), having experienced violence, reported that the violence had affected their job satisfaction. Qualitative data complemented this finding. While some described transient discomfort or difficulty meeting patients after the event, many described more profound effects, such as loss of empathy with patients. Several respondents described that the incident of violence had a radical impact on their subsequent career choices.

*“Maybe I am not brave enough to change my career but I definitely won't let any of my kids be a doctor…”* (Doctor, Bangladesh).

A joint display of quantitative and qualitative data for the impact of violence on physical and psychological health and job satisfaction is shown in Table 3.

**TABLE 3:** Rate and description of the physical and psychological consequences of experiencing workplace violence among 1016 providers of Obstetrics and Gynecology.

Categorical questions and response options		*n* (%)	Qualitative question and representative quotes	
Did the violence affect your physical health?	Yes	106 (13.9%)	*How did the violence affect your physical health?*	*“Migraines came after this event”*
	No	321 (42%)		*“Chronic pain is exhausting, I never hurt like I do now 3 years later”*
	No answer	337 (44.1)		*“Cardiac issues, insomnia, autoimmune disorder”*
				*“Tension headache, neck pain, gastritis, irritable bowel”*
Did the violence affect your psychological health?	Yes	362 (47.4%)	*How did the violence affect your emotional and psychological welbeing*?	*“Total isolation from everyone, over thinking frozen anxiety, panic attacks, can't think straight, depression. PTSD???”*
	No	65 (8.5%)		“*I lost sympathy with patients and their relatives. Lost interest in dealing patients. Felt disgusting. Cannot focus on professional work. Depression for the whole family.”*
				*“End of life.”*
	No answer	337 (44.1%)		*“Acute stress disorder. Persistent fears of similar patients, permanently.”*
Did the violence affect your job satisfaction?	Yes	222 (29.1%)	*How did the violence influence your job satisfaction or career choice?*	*“Maybe i am not brave enough to change my career bt i definitely won't let any of my kids be a doctor …”*
	No	204 (26.7%)		*“I retired from working in hospitals or clinics because when I went to do shifts, I had anxiety and panic attacks because of the fear of having to face all the upset relatives.”*
	No Answer	338 (44.2%)		*“At one point it made me think why I chose this profession where people are thankless and you never get any appreciation.”*

Abbreviation: *n* = number.

A little over half of the 764 providers (*n* = 438, 57.3%) who had experienced violence reported the incident; however, only 216 (28.3%) reported having received any kind of support. Qualitative analysis indicated that many respondents did not report the incident due to a feeling of resignation in knowing that nothing would be done, or a lack of recognition on their part that the incident deserved attention. Many respondents understood violence as “the risks of the job.”

*“Work with what pleases God. Trust in God. Do not ask about anything else.”* (OBGYN, Jordan, translated from Arabic).

Confirming our quantitative results, formal support structures were described as poor or lacking, with mainly informal support offered from colleagues.

*“I was abandoned by my director, only a support from my colleagues on the psychological and administrative level.”* (Doctor, Democratic Republic of the Congo, translated from French).

Only a minority of total respondents (*n* = 88/1016, 8.7%) had received training on WPV. Institutional policies on WPV were reported as effective or somewhat effective by 412 (40%) of respondents and not effective by 322 (31.7%). Qualitative data largely confirmed the absence of effective policies, with respondents often expressing powerlessness faced with their experience.

*“There is no law, no mechanism to handle any sort of abuse towards doctors. Once the dust settles you are supposed to chin up and return to your work like nothing happened.”* (OBGYN, Pakistan).

Two‐thirds of respondents (*n* = 654/1016, 64.4%) believed there should be stricter legal consequences for violence against medical providers. Many respondents cited the need for zero tolerance for abuse, including verbal abuse, against healthcare providers.

*“The ‘zero tolerance’ policy for violence against [healthcare providers] in the NHS is helpful and I believe does sometimes prevent violence, particularly physical violence.”* (Midwife, UK).

A joint display of data on support structures and training surrounding WPV is shown in Table 4.

**TABLE 4:** Support structures, workplace policies, training, and perceptions on legal repercussions for workplace violence among 1016 providers of obstetrics and gynecology.

Categorical questions and response options		*n* (%)	Qualitative question and representative quotes	
Did you report the incident?^a^	Yes	438 (57.3)	*Do you want to comment on reporting?*	*“We never talk about such incidences and consider them routine… so never think to report due to lack of awareness.”*
	No	259 (33.9)		
	No answer	67 (8.8)		*“I understood that these were the risks of the job.”*
Did you receive support?^a^	Yes	216 (28.3)	*Can you comment on the support you received?*	*“Thanks to Doctor, the organization supported me well.”*
	No	480 (62.8)		*“Work with what pleases God. Trust in God. Do not ask about anything else.”*
	No answer	68 (8.9)		
What type of support did you receive?^a^	Legal	41 (5.4)		*“I was abandoned by my director, only a support from my colleagues on the psychological and administrative level.”*
	Psychiatrist	7 (0.9)		
	Medical care	8 (1.0)		*“There is unfortunately a poor support system for healthcare professionals experiencing violence.”*
	Time off	16 (2.1)		
	Empathy	112 (14.7)		
How do you assess the effectiveness of workplace policies and training to prevent violence?	Very effective	165 (16.2)	*Can you comment on the workplace policies and measures?*	*“[Because of] corrupt and bad characters and background people in administration of the hospital, violence will not subside.”*
	Somewhat effective	247 (24.3)		
	Not effective	322 (31.7)		*“There is no law, no mechanism to handle any sort of abuse towards doctors. Once the dust settles you are supposed to chin up and return to your work like nothing happened.”*
	Do not know	27 (2.7)		*“Policies are in place but [people giving birth] and their support do not follow them and are not held accountable.”*
	No answer	255 (25.1)		
Have you received any training from your workplace to deal with violent situations?	Yes	88 (8.7)	*What kind of training did you receive?*	*“Training is about teaching to tolerate the violent and abusive behavior of patients and attendants. Training is not about correcting the circumstances that led to violent behavior. Training is about accepting or treating the fever but not treating the infection.”*
	No	606 (59.6)		
	No answer	322 (31.7)		*“Terrible online learning modules touting unproven theories designed by people who sit behind desks.”*
				*“How to ‘deescalate’—yeah, that works! 🤣🤣🤣”*
Do you believe that there should be stricter legal consequences for violence against healthcare professionals?	Strongly agree	516 (50.8)	*What would you like to see in your workplace to help healthcare professionals cope with violence?*	*“There should be medicolegal lawyers to defend in every healthcare provider organization.”*
	Agree	138 (13.6)		
	Neutral	23 (2.3)		*“The ‘Zero tolerance’ policy to violence against healthcare professionals in the NHS is helpful and I believe does sometimes prevent violence, particularly physical violence.”*
	Disagree	4 (0.4)		
	Strongly disagree	1 (0.1)		*“We need a strong law to protect us.”*

^a^ Asked of the 764 respondents who reported having experienced workplace violence.

The presence of triggers or contributing factors for the violence was asked of all 1016 respondents, whether or not they had personally experienced violence, and they answered as follows in order of frequency: staff shortages (*n* = 394, 38.8%); lack of security personnel (*n* = 381, 37.5%); long waiting times (*n* = 379, 37.3%); staff attitudes (*n* = 294, 28.9%); perpetrator mental health issues (*n* = 262, 25.9%); genuine medical negligence (*n* = 194, 19.1%); and substance abuse on the part of the perpetrator (*n* = 108, 10.6%).

Confirming the categorical data, many respondents described that insufficient infrastructure, staffing, and resources contributed to abuse against providers. Complementary text data showed that respondents recognized that fear, pain, and desperation on the part of patients or relatives were catalysts to violence and resulted from lack of knowledge or unrealistic expectations about what providers could achieve. Several respondents also described a pervasive distrust of medical providers, exacerbated by social media, that led to a climate of fear and antagonism between patients and providers.

*“Bad reception upon arrival. Husband's fatigue: patient having been referred from another health facility. Prejudices about the staff.”* (OBGYN, Côte D'Ivoire, translated from French).

A joint display of findings on triggers for violence against providers is shown in Table 5.

**TABLE 5:** Rate and description of perceived triggers of workplace violence among 1016 providers of obstetrics and gynecology.

Categorical questions and response options			*n* (%)	Qualitative question	Text responses, samples
What triggers for workplace violence do you believe exist?	Genuine medical negligence	Yes	194 (19.1)	*Can you describe potential triggers of violence in your workplace?*	*“Patients who did not understand doctors' decisions either through miseducation by third parties or lack of education.”*
		No answer	822 (80.9)		
	Mental health issues—patient	Yes	262 (25.9)		*“Bad reception on arrival. Husband's fatigue: patient having been referred from another health facility. Prejudices about the staff.”*
		No answer	753 (74.1)		
	Substance abuse disorder—patient	Yes	108 (10.6)		*“Pain levels, long wait times, unmet expectation and disagreements.”*
		No answer	908 (89.4)		*“Financial problems.”*
	Long waiting times	Yes	379 (37.3)		*“I think the overall negative picture that's being painted in the social media about doctors makes people very suspicious and untrusting when it comes to doctors.”*
		No answer	635 (62.5)		
	Lack of security personnel	Yes	381 (37.5)		*“Her expectations were higher than what I could offer.”*
		No answer	635 (62.5)		*“Impotence of patients' own clinical situation. Not facing and accepting the illness.”*
	Staff shortages	Yes	394 (38.8)		
		No answer	622 (61.2)		*“Probably the pain or despair of the patients.”*
	Staff attitudes	Yes	294 (28.9)		*“Poverty. Ignorance. Already in belief that when they reach the hospital it is doctor's duty to do everything.”*
		No answer	722 (71.1)		

## 4 DISCUSSION

### 4.1 Main findings

We found that WPV against OBGYN providers is pervasive across settings, with young and junior providers being most at risk, and that victims experience wide‐ranging and long‐lasting physical and psychological repercussions that affect performance, satisfaction, and retention to the profession. WPV seems to be triggered by a complex interplay of factors, and insufficient structures and policies exist to prevent violence and support those affected.

All previous studies indicate that WPV against healthcare workers as a group is common and likely underreported, with 1‐year incidence rates of verbal and physical violence in the range of 33.8%–78% and 8.5%–31%, respectively, across five systematic reviews.^3^, ^8^, ^11^, ^12^, ^13^ Several of these studies further confirm our finding that young or less experienced providers are more exposed to violence, and that being male and a nurse is associated with a higher risk of physical violence, which our study did not find.^3^, ^6^, ^12^, ^13^ That our senior providers reported lower rates of lifetime WPV experience may seem counterintuitive as older doctors have more years in which to experience violence. It is possible that older respondents have forgotten the violence they experienced when they were new on the job. It is also possible that violence towards doctors is increasing and junior doctors are at the frontline of an environment where violence has become more prevalent. Several studies and systematic reviews indicate that this is the case, with WPV further exacerbated during the recent COVID pandemic.^5^, ^7^, ^14^, ^15^

What our study adds to the current evidence is a characterization of WPV both in terms of what the experience consisted of and its repercussions. Our results indicate that even verbal abuse can lead to complex, long‐lasting, and severe mental distress. Previous research on violence suggests a distinction between affective and predatory violence, with the latter being more detrimental for the victim. Affective violence arises from of a “fight or flight” response, which, in the context of medical care, is often fear or pain. In contrast, predatory violence is an intentional and deliberate attack motivated by a grievance. Consistent with this, many respondents recognized that threats to their person and accusations of mismanagement and incompetence had particularly painful and had long‐lasting effects.^16^ A type of violence that emerged in our data, which has not been previously reported, was the threat of leveraging social media to discredit providers. The Internet is a source of legitimate information about the quality of services, which makes this type of abuse more difficult to categorize. Nevertheless, workplace safety policies must recognize the illegitimate use of social media as another form of violence against providers, be prepared to counter personal abuse that emanates from social media platforms, and redirect criticisms of services to legitimate channels.

We also found indications that, although respondents expressed understanding for factors that triggered the violence, their empathy and engagement with patients and similar clinical scenarios sometimes changed permanently for the worse. This study further highlights the complex setting in which WPV in healthcare settings occurs, in that both the aggressor and the victim are under duress. The stress of an untreated emergency, the unrecognized pain of a loved one, and navigating labyrinthine and unaccountable healthcare systems are easily understood to heighten feelings of desperation and anger. At the same time, healthcare providers may be operating under the extreme pressure of insufficient time and resources to meet patient needs. Our data support a prevalent atmosphere of distrust towards providers fueled by unrealistic or unmet expectations. In both the USA and India, providers increasingly report fearing for their safety and the use of weapons or private security staff has become more prevalent.^14^, ^17^

Almost two out of five providers in our study believed their hospital's policies were, to some degree, effective in preventing and managing WPV. However, a recent Cochrane review concludes that although a wealth of interventions exist to mitigate WPV, their focus is primarily on de‐escalation and there is no evidence that they work to reduce the occurrence, or severity, of WPV.^1^ As previous authors have noted, there is a lack of evidence‐based research on successful measures to prevent or mitigate WPV.^14^ Most studies indicate that support structures are not only ineffective, but lacking.^18^ A recent systematic review of 17 studies on the prevention of WPV found that training improved providers' perceived ability to handle violent events but no study measured the interventions' effect on the incidence of WPV or its repercussion for the provider.^19^ A recent study found a strong association between a hospital having solid policies on workplace safety, and having solid policies on patient safety.^20^ This mirrors our finding that WPV often occurs in a context that is perceived as unsafe for both patients and providers. Tolerance for long waiting times or poor outcomes are likely higher in a clinical setting in which the patient and their relatives trust the institution providing care. The FIGO Committee has developed a tool kit to prevent and mitigate WPV against OBGYN, the development process of which will be described in another article.

It is likely that addressing violence against healthcare providers requires a comprehensive approach involving healthcare institutions, policymakers, law enforcement agencies, healthcare professionals themselves, and the broader community.

### 4.2 Strengths and limitations

To our knowledge, this is the first global survey on WPV among OBGYN providers and the only study examining the prevalence and character of WPV also through a qualitative lens. Using a retrospective survey as data measurement risks both selection bias, in that people affected by violence are more likely to respond, and recall bias, in asking people to remember events far in the past. The collection of qualitative data through a survey, unlike in an interview setting, did not allow for individualized follow‐up questions, which could have nuanced the responses and the quality of the data. Nevertheless, the quantity, detail, and depth of our text responses suggest that the experience of WPV and its repercussions are not easily forgotten.

## 5 CONCLUSION

Our findings suggest that WPV against OBGYN providers is frequent across settings, and that victims experience wide‐ranging and long‐lasting physical and psychological repercussions that affect performance, satisfaction, and retention to the profession. WPV may be triggered by a complex interplay of factors and insufficient structures and policies exist to prevent violence and support those affected.

## AUTHOR CONTRIBUTIONS

R.S. had the idea for the study and led study procedures. R.S. designed the survey that was revised by M.E. and A.R. and approved by the members of the Committee on Women Facing Crises. M.E. analyzed the data and wrote the draft for the manuscript. All authors have approved the final version of the manuscript.

## CONFLICT OF INTEREST STATEMENT

The authors have no conflicts of interest.

## MEMBERS OF THE FIGO COMMITTEE ON WOMEN FACING CRISES, 2023–2025

Atziri Ramirez (Chair), Rubina Sohail, Lubna Hassan, Margit Endler (Vice‐chair), Denis Mukwege, Miguel Gutierrez, Anwar Marjan, Kristina Jariene, Priyankur Roy, Ana Patricia Gomez, Sarah Baffoe, Diana Galimberti, Shantha Kumari.

## Supporting information


Data S1.

## Data Availability

The data that support the findings of this study are available on request from the corresponding author. The data are not publicly available due to privacy or ethical restrictions.
