# Supplementary material for: A silent pandemic of violence against providers in obstetrics and gynecology: A mixed‐methods study based on a global survey
Source: Int J Gynaecol Obstet. 2024 Nov 2;168(1):377–86. doi: 10.1002/ijgo.15985 (PMC11649870; doi:10.1002/ijgo.15985)
Supplement: Supplementary file 1 — Data S1. [file IJGO-168-377-s001.docx]

**Supplementary Table A** List of countries by regional categorization from where respondents answered a survey on violence against providers of obstetrics and gynecology (n = 1016)

| Africa | Cote d’Ivoire, Democratic Republic of the Congo, Egypt, Ethiopia, Ghana, Kenya, Malawi, Morocco, Namibia, Niger, Nigeria, Rwanda, South Africa, South Sudan, Tanzania |
| --- | --- |
| Australasia | Australia |
| Central Asia | Afghanistan, Kashmir, Pakistan |
| South and East Asia | Bangladesh, Bhutan, Hong Kong, Japan, India, Indonesia, Mongolia, Myanmar, Nepal, Philippines, Sri Lanka, Taiwan |
| Europe/Russia | Austria, Denmark, France, Georgia, Germany, Greece, Ireland, Italy, Latvia, Lithuania, the Netherlands, Norway, Portugal, Romania, Russia, Serbia, Spain, Sweden, Switzerland, United Kingdom |
| Middle East | Iran, Iraq, Jordan, Kuwait, Lebanon, United Arab Emirates |
| North America | Canada, United States of America |
| South and Central America | Argentina, Bolivia, Brazil, Chile, Colombia, Dominican Republic, Ecuador, El Salvador, Guatemala, Guyana, Mexico, Panama, Paraguay, Peru, Venezuela |
